# The *TBXT* rs2305089 SNP links the benign notochordal cell tumour and chordoma

**DOI:** 10.1002/path.6427

**Published:** 2025-05-05

**Authors:** Inga Usher, Paul O'Donnell, Lorena Ligammari, Dorothee Harder, Wendy Brown, David Choi, Paul Cool, Lucia Cottone, Adrienne M Flanagan

**Affiliations:** ^1^ Department of Pathology University College London Cancer Institute London UK; ^2^ Victor Horsley Department of Neurosurgery The National Hospital for Neurology and Neurosurgery London UK; ^3^ Department of Musculoskeletal Radiology Royal National Orthopaedic Hospital Stanmore UK; ^4^ Department of Cancer Biology University College London Cancer Institute London UK; ^5^ Department of Radiology University Hospital Basel Switzerland; ^6^ Department of Radiology Royal Prince Alfred Hospital Sydney Australia; ^7^ Department of Orthopaedic Surgery Robert Jones and Agnes Hunt Orthopaedic Hospital Gobowen UK; ^8^ Keele University Keele UK; ^9^ Department of Histopathology Royal National Orthopaedic Hospital Stanmore UK

**Keywords:** benign notochordal cell tumour, chordoma, BNCT, SNP, notochord, spine, TBXT, lysosome, iPSC

## Abstract

The aim of this research was to investigate the pathogenesis of the bone cancer chordoma and the role of the germline rs2305089 SNP in *TBXT*. Using medical imaging and genotyping studies, we observed that benign notochordal cell tumours (BNCTs) were associated with chordomas and with the variant rs2305089 A‐allele with enrichment of the AA genotype compared to controls. We engineered *in vitro* mesoderm models, representing notochord, which showed higher expression of *TBXT* and activation of its regulatory network in the presence of the variant A allele. Heterozygotes (GA) displayed enrichment of Wnt/β‐catenin and epithelial mesenchymal transition pathways, faster cell migratory capacity, and altered expression of endoplasmic reticulum and intracellular transport mediators. WT lines (GG) were enriched for metabolic pathways and MTORC1 signalling, suggesting that rs2305089 genotype regulates notochord vacuoles during notochord regression. By leveraging patient‐derived data and functional studies, we show that the variant rs2305089 A‐allele predisposes to BNCTs and ultimately to chordomas. © 2025 The Author(s). *The Journal of Pathology* published by John Wiley & Sons Ltd on behalf of The Pathological Society of Great Britain and Ireland.

## Introduction

Chordomas are rare primary tumours of the skull base, spine, and sacral‐coccygeal bones [1]. Surgery and radiotherapy are the standard of care as there are no FDA‐approved therapeutic agents. Some (40%) of these tumours metastasise, and the median survival of 7 years has not improved significantly in several decades [2]. Chordomas show notochordal differentiation and are hypothesised to arise from the notochord [3, 4], which is the defining feature of the chordate phylum. During human embryonic development, the notochord is the precursor of the axial skeleton (skull base and spine): Under physiological conditions it forms from the mesoderm during the first trimester [5] and regresses to become the nucleus pulposus of the intervertebral discs before birth. The mechanisms underlying human notochord regression are incompletely understood. Notochord regression occurs in rats, by apoptosis [6], but not in mice [7] and can be incomplete. Islands of persistent notochordal cells, known as benign notochordal cell tumours (BNCTs) [8], have been found in the vertebrae of humans and mice [9, 10]. However, the pathogenesis of chordoma has not been fully elucidated.

BNCTs are present in up to 20% of the general population [8], while chordomas affect 1 in 800,000 people per year, indicating that if BNCTs were the precursors of chordomas, the conversion to malignant disease is low [11]. In clinical practice, BNCTs are managed with radiological surveillance without a consensus regarding  duration or frequency [12]. Numerous reports document the co‐existence of BNCTs and chordomas, implying that a chordoma develops from the malignant transformation of a BNCT [8, 13, 14], but no longitudinal studies have demonstrated this definitively, and it has been argued that their co‐existence could be coincidental [12, 15, 16].

The notochord, BNCTs, and chordomas express the transcription factor TBXT (also known as brachyury), encoded by *TBXT* [4, 13, 17]. *TBXT* duplication has been reported in up to 27% of sporadic chordomas [18, 19, 20], and *TBXT* is the top selectively essential gene out of ~18,000 genes in chordoma cell lines and patient samples [21]. The *TBXT* locus is associated with a super enhancer [21, 22, 23], and degradation of the transcriptional condensate at this locus causes chordoma cells to enter senescence [23], as does TBXT knockdown [24], underscoring the dependence of chordomas on *TBXT*. Beyond *TBXT* and *CDKN2A*, recurrent genetic alterations are uncommon but affect *LYST*, a lysosomal trafficking regulator, and chromatin remodellers, particularly *PBRM1*. It is noteworthy that ~50% of chordomas lack a driver mutation [19, 20, 25], highlighting the need to better understand their biology.

The most common genetic alteration associated with both familial and sporadic chordomas is the rs2305089 germline SNP, with which there is an exceptionally strong association: 97% of patients with sporadic chordoma harbour at least one variant allele (A) [26, 27]. This missense SNP results in the substitution of an uncharged amino acid for an acidic residue (Glycine 177 for Aspartic Acid, G177D) in exon 4, close to the DNA binding domain of TBXT, affecting its thermostability [28], dimerization, and binding to DNA *in vitro* [29]. *TBXT* expression is higher in patients with the homozygous variant (AA) than the heterozygous genotype (GA) [26]. TBXT acts on a network of over 600 genes in chordomas encompassing cell cycle regulators, producers of extracellular matrix, and growth factors [24], making it likely that these play a role in the pathogenesis of chordomas, but the functional impact of the rs2305089 SNP has yet to be elucidated. To date, the rs2305089 SNP has not been linked to BNCTs.

Here, we investigated the development of chordomas from the notochord and BNCTs. First, we established an association between BNCTs and chordomas and with the rs2305089 variant A allele. We then employed CRISPR/Cas9 technology and a stem cell‐derived mesoderm model to investigate the functional impact of this SNP on notochordal differentiation and predisposition to chordoma.

## Materials and methods

### Patient samples and data

Samples were collected under the Royal National Orthopaedic Hospital (RNOH) Biobank, approved by the National Research Ethics Committee of the Health Research Committee (reference: Integrated Research Application System (IRAS) project identifier: 272816). This study was approved by the National Research Ethics Committee approved UCL/UCLH Biobank Ethics Committee (project no: EC17.14). Samples were anonymised using Pro‐Curo software (Pro‐Curo Software Ltd., Horsham, UK).

### Medical imaging study

MRI was assessed by clinically certified radiologists with expertise in musculoskeletal disease (POD, DH, WB) to establish the prevalence of BNCTs. This was a retrospective study using images generated using different scanners. Patients were included if sufficient sequences were available to determine the signal characteristics of a lesion: low T1 intensity, high T2 intensity, and short tau inversion recovery (STIR) sequences, well defined, confined to bone/intramedullary without adjacent marrow oedema, and with occasional punctate fat. Significant contrast enhancement or soft tissue extension favoured a chordoma [12].

#### Inclusion criteria

Healthy control cohort: Patients without cancer with whole spine MRI (January 2015–January 2022) (supplementary material, Table S1). As the cohort of whole‐spine MRIs (*n* = 171) was predominantly female, we included all male patients (*n* = 51) and randomly selected female patients (*n* = 23) from the remaining list to ensure the sex and ethnicity proportions reflected the chordoma group.

Chordoma patients: Patients with a tissue diagnosis of chordoma with MRI of any section of their spine as whole‐spine imaging is not standard practice for new chordoma diagnoses. The founder cohort (*n* = 109) was identified at the RNOH, validation cohort 1 (*n* = 23) from University Hospital Basel, Switzerland, and validation cohort 2 (*n* = 14) from the Royal Prince Alfred Hospital, Sydney.

### Sample size calculation

See details in Supplementary materials and methods.

### TaqMan genotyping of the rs2305089 locus

Rs2305089 genotyping was performed as described in Supplementary materials and methods on patients identified in the radiological study as having a BNCT with and without chordoma. The control group were patients without a BNCT or chordoma for whom germline DNA was available (separate from the control patients in the radiological study for whom DNA was not available).

### Induced pluripotent stem cell‐derived mesoderm‐like cell cultures

A viral‐integration‐free human induced pluripotent stem cell (iPSC) line generated from cord blood‐derived CD34+ progenitors was obtained from Gibco™/Thermo Fisher Scientific (Catalogue No.: A18945) and grown in serum‐free culture conditions following the manufacturer's instructions [30] (see Supplementary materials and methods).

For mesoderm differentiation, iPSCs were subcultured into multiwell plates coated with Geltrex and grown for 12 h, ensuring sufficient space for colonies to grow from single cells. Colonies were recovered overnight in E8 Flex medium. For each cell line, three biological replicates were plated (three individual mesoderm differentiation inductions, one per well) for each time point: 0, 24, 48, 72 h (schema in Figure 2B). Twenty‐four hours after seeding, cells were either collected at the 0‐h time point or differentiated to mesoderm by adding Cardiomyocyte Differentiation Medium A (Thermo Fisher Scientific, A29209‐01), which was refreshed 24 h later (24‐h time point). At the 48‐h time point cells were either collected or the medium was changed to Mesenchymal Stem Cell Growth Medium 2 (MSC2, PromoCell, C‐28009) for MSC maintenance, incubated for another 24 h, and collected at the 72‐h time point. Attempts to expand cells in the mesoderm phase resulted in widespread cell death. A preliminary experiment assessing *TBXT* expression at early time points (0, 8, 16 h) showed no expression at 8 h and no difference between genotypes at 16 h, so these data are not shown.

### CRISPR/Cas9 genetic editing

To genetically replace a single nucleotide in iPSCs using CRISPR/Cas9, we followed the protocol in Supplementary materials and methods and in [35]. Single guides and single‐stranded oligonucleotide donor templates are reported in supplementary material, Table S3 and Figure S1B.

### Sanger sequencing and digital droplet PCR (ddPCR)

Gene editing outcomes were assessed using Sanger sequencing and ddPCR [35] (Supplementary materials and methods). Primers are listed in supplementary material, Table S3.

### Quantitative real‐time PCR (qPCR) and western blotting

Methods for qPCR are described in Supplementary materials and methods and primers listed in supplementary material, Table S3. Western blotting is described in Supplementary materials and methods [31].

### Messenger RNA (mRNA) sequencing

#### Samples and library preparation

Cells were prepared and RNA was extracted as described in Supplementary materials and methods. Libraries were prepared using TruSeq Stranded mRNA Library Prep kit (Illumina). Each sample underwent 20 million 2 × 100 paired read sequencing on the Illumina NovaSeq.

#### mRNAseq processing and analysis

Raw read quality was assessed with FastQC (version 0.11.5) aligned to GRCh38 with Salmon [36] using default options. Differential gene expression analysis was performed using DESeq2 version 1.36.0 using cell line/genotype as factors [37]. Differentially regulated genes were based on >1.5 log2‐fold‐change and *p* value < 0.05. Principal component analysis was performed on variant‐stabilising transformed data using the plotPCA function in the DESeq2 package.

#### Gene set enrichment analysis of direct and indirect targets of TBXT

Gene set enrichment analysis (GSEA) was performed using directly and indirectly regulated targets of TBXT reported in [24].

### Functional assays

Apoptosis and cell cycle studies were performed as described in Supplementary materials and methods and reported previously [22] at 48 h during the mesoderm phase when the expression of *TBXT* was different between genotypes. Each assay was repeated twice in duplicate (two biological replicates/cell line).

#### Monolayer wound/scratch migration assay

iPSCs were seeded in 96‐well Geltrex‐coated plates (five replicate wells per cell line) in 250 μl E8 Flex medium with a 1:14 splitting ratio. At 50–60% confluence, 200 μl Cardiomyocyte Differentiation Medium A was added. After 24 h, when cells were 95% confluent, wounds were created using the Incucyte® 96‐Well Woundmaker Tool, washed once with Cardiomyocyte Differentiation Medium A, and plates were scanned for 22 h using an Incucyte™ SX5 live cell imaging system (Essen BioScience, Ann Arbor, MI, USA) with four pictures every 2 h to measure scratch closure.

### Statistical analysis

Statistical analysis was performed using GraphPad Prism version 10.2.2 (GraphPad Software, Boston, MA, USA) and R version 4.2.1. *P* values < 0.05 were considered significant. Statistical parameters including the exact value of *n* and statistical significance are reported in the figures and figure legends. Precision measures are reported as mean ± SD unless otherwise specified to be mean ± standard error of the mean (SEM). In figures, asterisks denote statistical significance: *p ≤ 0.05, **p ≤ 0.01, ***p ≤ 0.001, ****p ≤ 0.0001. For the GSEA a false discovery rate (FDR) <0.25 was considered significant.

## Results

### BNCTs and chordomas are associated

**Figure 1:**
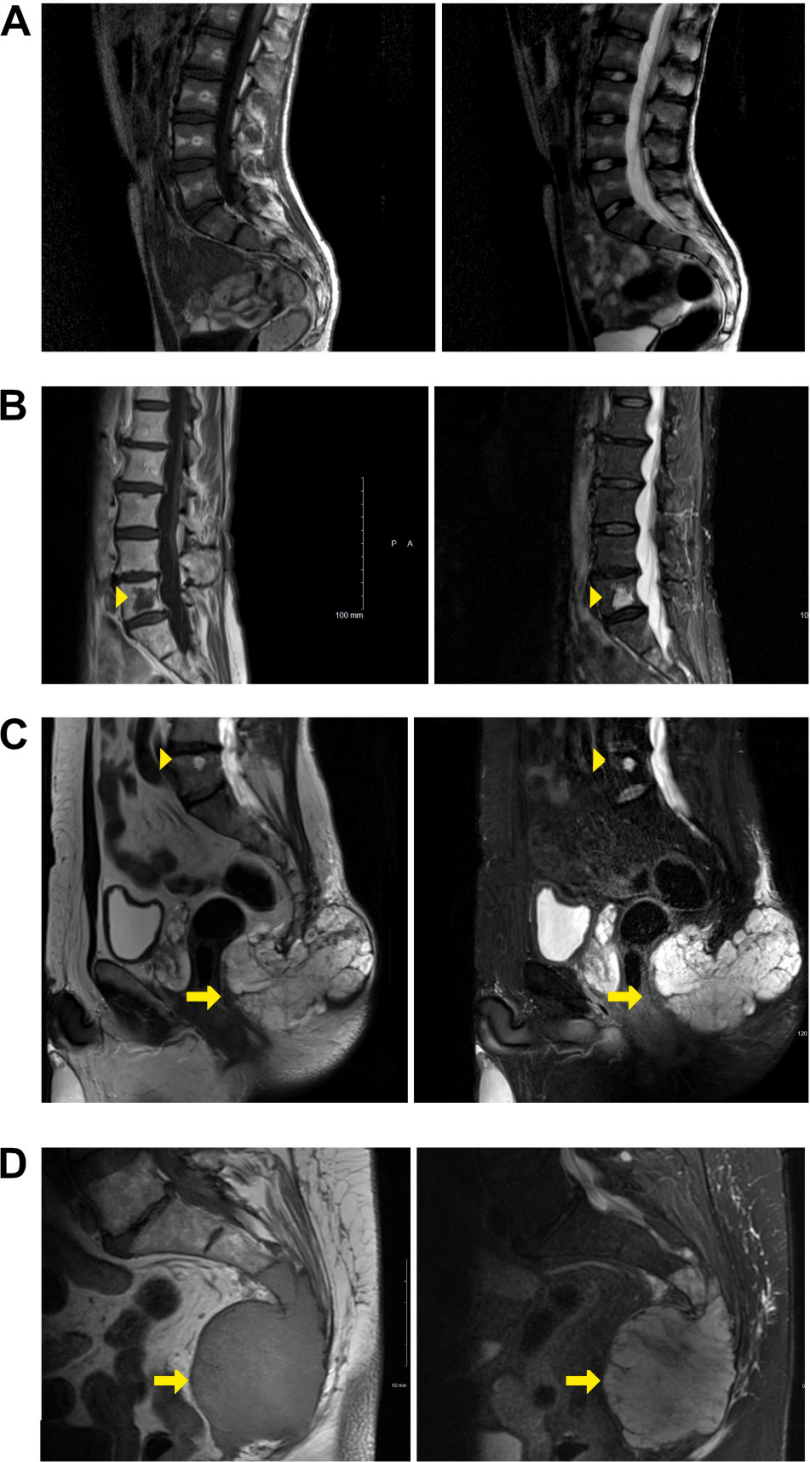
Representative spinal MRI scans of study groups. T1 images are shown on left of each panel and T2 or STIR images on right of each panel, except for C, which shows a T2 and a STIR image. Arrowheads indicate BNCTs, and arrows indicate chordomas. (A) Control group: no BNCT or chordoma on MRI. (B) BNCT group: BNCT of fifth lumbar vertebra showing typical signal characteristics and confinement to bone. (C) Chordoma with coexisting BNCT group: sacrococcygeal chordoma and BNCT in fifth lumbar vertebra. (D) Chordoma‐only group: sacrococcygeal chordoma.

We first asked whether there was an association between BNCTs and chordomas by comparing the radiological prevalence of BNCTs on spinal MRI from 109 patients with a chordoma and a control group of 74 patients without cancer (Figure 1). The study groups are summarised in supplementary material, Table S1. The most common reasons for undergoing MRI in the control group included scoliosis (25/74; 34%), previous spinal surgery (23/74; 31%), degenerative conditions (14/74; 19%), and spinal cord injury (13/74; 18%).

One BNCT was identified in 3% (2/74) of the healthy control group (Table 1). In contrast, 16/109 (15%) patients with a chordoma were found to have at least one BNCT (*p* = 0.001), 6/109 (6%) harboured more than one BNCT (*p* = 0.003), and all BNCTs were anatomically separate from the chordoma, implying a systemic predisposition for developing a BNCT (Table 1). The mean age of patients with and without a BNCT in the chordoma cohort was 64 years and 53 years respectively (*p* = 0.052).

**Table 1:** Prevalence of BNCTs on MRI scans of patients in control group and chordoma group at three orthopaedic centres.

	Control group	Founder cohort	Validation cohort 1	Validation cohort 2
Total no. MRI scans	74	109	23	14
No. patients with one BNCT (%)	2 (3)	16 (15)	6 (26)	4 (29)
*p* value versus control group	‐	0.001	0.002	0.005
No. patients with >1 BNCT (%)	0 (0)	6 (6)	0	0
*p* value versus control group	‐	0.003		
No. MRIs that covered whole spine* (%)	74 (100)	27 (25)	2 (9)	Not known

*P* values were calculated using Fisher's exact test.

* Whole‐spine MRI included the coccyx in 25/74 (34%) patients in control group.

In two validation cohorts from other centres, the proportion of patients who harboured a coexistent BNCT and chordoma was similar to that in the founder cohort (*p* = 0.233). However, in the validation cohorts, only patients with one BNCT were identified, and this was considered likely to be due to the small number of patients (Table 1). These results demonstrate an association between BNCTs and chordomas.

### The rs2305089 variant A allele is associated with BNCTs

Next, we analysed the rs2305089 genotype in germline DNA from a cohort of 33 patients with a BNCT, 13 patients with both a chordoma and a BNCT, and 132 patients without either lesion (Table 2). Samples from a previous study that had undergone whole genome sequencing [19] were used to validate the genotype calls.

**Table 2:** Genotypes of rs2305089 SNP in study groups.

Group	VAF	AA	GA	GG	Number of patients	*p* value
BNCT	0.71	16	15	2	33	0.002
Concurrent BNCT and chordoma	0.85	9	4	0	13	<0.001
Control (no BNCT or chordoma)	0.49	32	66	34	132	‐

*P* values were calculated using Fisher's exact test compared to control group.

AA, homozygous for chordoma‐associated variant allele; GA, heterozygous; GG, homozygous for ancestral allele; VAF, variant allele frequency.

The variant allele frequency (VAF) of the control group (0.49) concurred with what was reported previously (0.47–0.51) [26, 27]. The rs2305089 variant A allele, and specifically the homozygous variant AA genotype, was enriched in patients with a BNCT compared to controls (*p* = 0.002), suggesting that the BNCT and the SNP were associated (Table 2). Furthermore, the rs2305089 VAF in the BNCT group (0.71) was not statistically different (*p* = 0.064) to that of patients with chordomas reported in the literature (0.75–0.86) [26, 27], supporting the concept that the BNCT is the precursor of chordoma.

In conclusion, we confirm that the rs2305089 variant A allele is strongly associated with chordoma and show for the first time that it is also associated with the BNCT.

### Establishment of an isogenic model to study the role of the rs2305089 SNP during mesoderm/notochord differentiation

**Figure 2:**
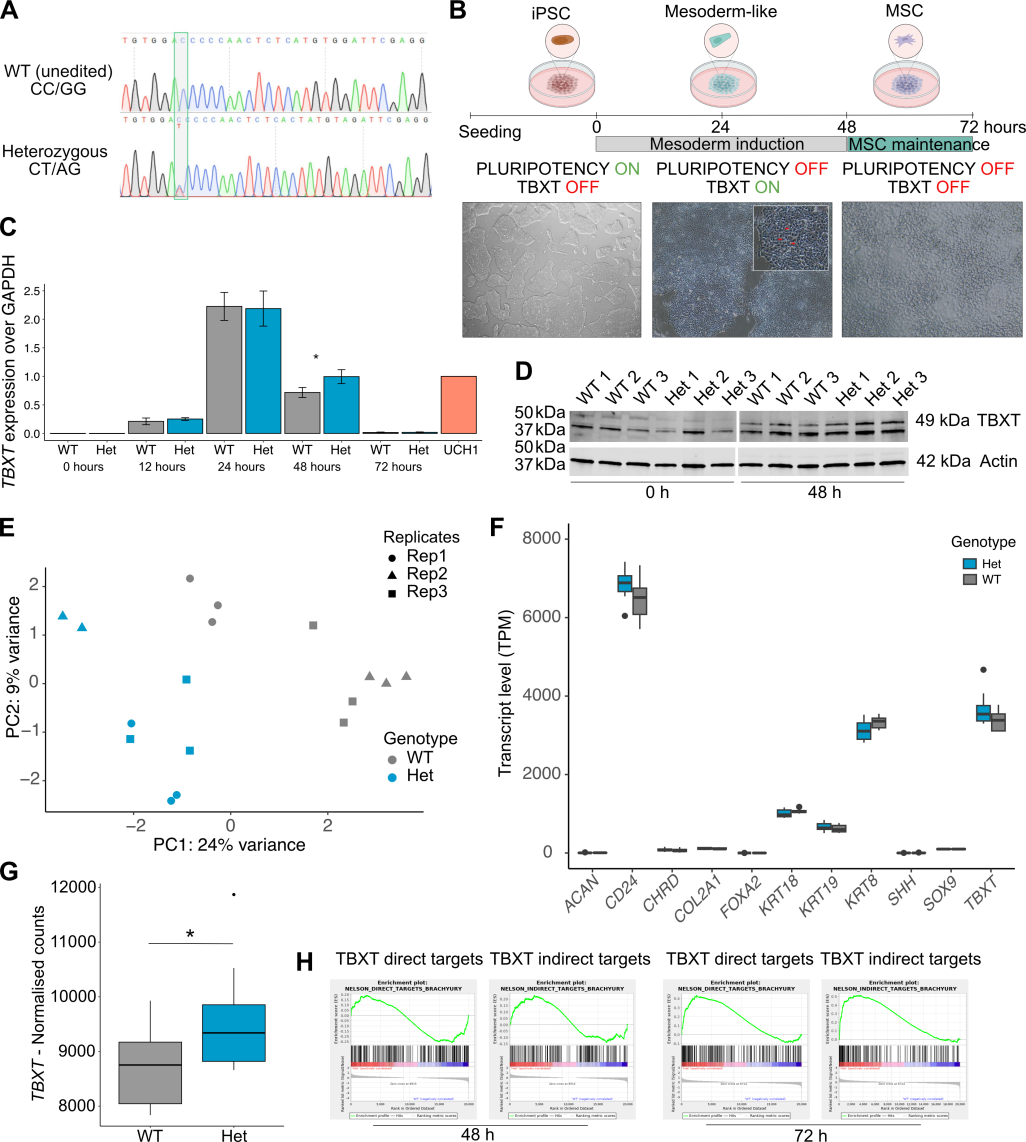
The rs2305089 SNP impacts expression of TBXT and its target genes in a model of iPSC‐derived mesoderm. (A) Representative Sanger sequencing traces of edited iPSC lines. (B) Cartoon of differentiation protocol: iPSC lines were differentiated into mesoderm over 48 h, then into MSCs between 48 and 72 h. Bottom: representative bright field images of WT cell line subjected to differentiation (×10 magnification). At 48 h the mesodermal cells adopted a vacuolated appearance, as shown by red arrows in the insert (×40). Created with Biorender.com. (C) Expression of TBXT by qPCR. Three cell lines, three biological replicates per time point. The U‐CH1 chordoma cell line, which expresses TBXT, was used as a positive control. *p* = 0.002, two‐way ANOVA. (D) Western blots for TBXT (top) and β‐actin (bottom) in the three WT and Het cell lines cultured in iPSC medium (0 h) and in mesoderm differentiation medium for 48 h. For TBXT, the lower band is known to be non‐specific [31]. TBXT was absent in the iPSC state for all cell lines and was expressed only upon mesoderm induction; Het cell lines expressed higher levels of TBXT at 48 h compared to WT. The blots were cut for incubation and images aligned in the panel shown. (E) Principal component analysis of all mRNAseq data. Cell lines (*n* = 3 per genotype) and replicates (*n* = 3 per cell line except Het2 replicate 2, which did not pass quality control due to a low read count) were collapsed into genotypes. mRNAseq was undertaken at 48 h, shown here, and at 72 h, shown in supplementary material, Figure S2D. (F) Expression of 11 putative notochord markers [21, 32, 33, 34] in mRNAseq data at 48 h. Five markers were expressed by all mesoderm cells. Three biological replicates per genotype. TPM = transcripts per million. (G) Expression of *TBXT* in Het and WT cell lines at 48 h (mRNAseq). *p* = 0.01, Wilcoxon test. Three cell lines and three biological replicates per genotype. (H) Gene set enrichment analysis (GSEA) of direct and indirect targets of TBXT from its gene regulatory network [24]. Enrichment plots of TBXT targets after 48 h of differentiation (FDR = 0.42 direct, FDR 0.61 indirect) and at 72 h (FDR = 0.004 direct, FDR 0 indirect). Het cell lines on the left (in red, positively correlated) and WT cell lines on the right (in blue, negatively correlated) in each plot. Three cell lines and three biological replicates per genotype.

We next asked if the variant A allele predisposes to BNCT by altering the *TBXT* gene regulatory network during mesoderm/notochord differentiation. To address this, we engineered iPSC isogenic clones that differed only by their genotype at the rs2305089 locus using CRISPR homology‐directed repair (knock‐in) (Figure 2A and supplementary material, Figure S1). Several heterozygous (Het, GA alleles) and homozygous unedited (WT, GG alleles) clonal cell lines were isolated, and six were taken forward (supplementary material, Table S2). No homozygous (AA) knock‐in lines were generated, confirming the difficulty of editing both alleles reported by others [23]. No off‐target modification was detected around the *TBXT* gene (1‐kb region, supplementary material, Data S1 and S2).

To test how the rs2305089 A allele influenced the expression of *TBXT*, we exploited a previously optimised model of iPSC‐derived mesoderm‐like cells [22] (Figure 2B). Upon mesoderm induction, *TBXT* expression rose rapidly, peaked at 24 h, and remained comparable to the U‐CH1 chordoma cell line at 48 h (Figure 2C). From mesoderm, the same cells were differentiated into mesenchymal stem cells (MSCs), and *TBXT* expression fell to undetectable levels by 72 h (Figure 2C). This expression pattern of *TBXT* matches that of notochord‐like cells from human and murine ESCs [32, 38], supporting the relevance of this model. Of note, we found no evidence for allele‐specific expression of the A allele, corroborating previous findings [28], to explain why individuals who are heterozygous for the SNP are predisposed to developing BNCT and chordoma (supplementary material, Figure S2A).

### The rs2305089 SNP influences expression of *TBXT* and its gene regulatory network

As the variant A allele is associated with significantly increased *TBXT* expression in chordomas [26], we next asked if the presence of the variant A allele influenced *TBXT* expression in our cell models. *TBXT* expression by qPCR was similar across all cell lines at 24 h, but expression was found to be higher in heterozygous compared to WT cell lines at 48 h (Figure 2C and supplementary material, Figure S2B); this was confirmed at the protein level (Figure 2D and supplementary material, Figure S2C).

We performed bulk RNA sequencing (mRNAseq) when *TBXT* expression peaked at 48 h (Figure 2E and supplementary material, Data S3) and at 72 h when effects on downstream pathways might be expected (supplementary material, Figure S2D and Data S3). Samples clustered by genotype as expected at both time points (Figure 2E and supplementary material, Figure S2D). All cell lines expressed previously reported putative notochord markers [21, 32, 33, 34] (Figure 2F). Heterozygous cell lines showed higher expression of *TBXT* (Figure 2G and supplementary material, Figure S2B) and its direct and indirect targets [21, 24] compared to WT lines (Figure 2H and supplementary material, Data S4, FDR 0.004 and 0). We conclude that rs2305089 variant A allele plays a role in the persistence of notochordal cells by modulating the expression of *TBXT* and its network.

### The rs2305089 SNP influences global gene expression and the hallmarks of cancer

**Figure 3:**
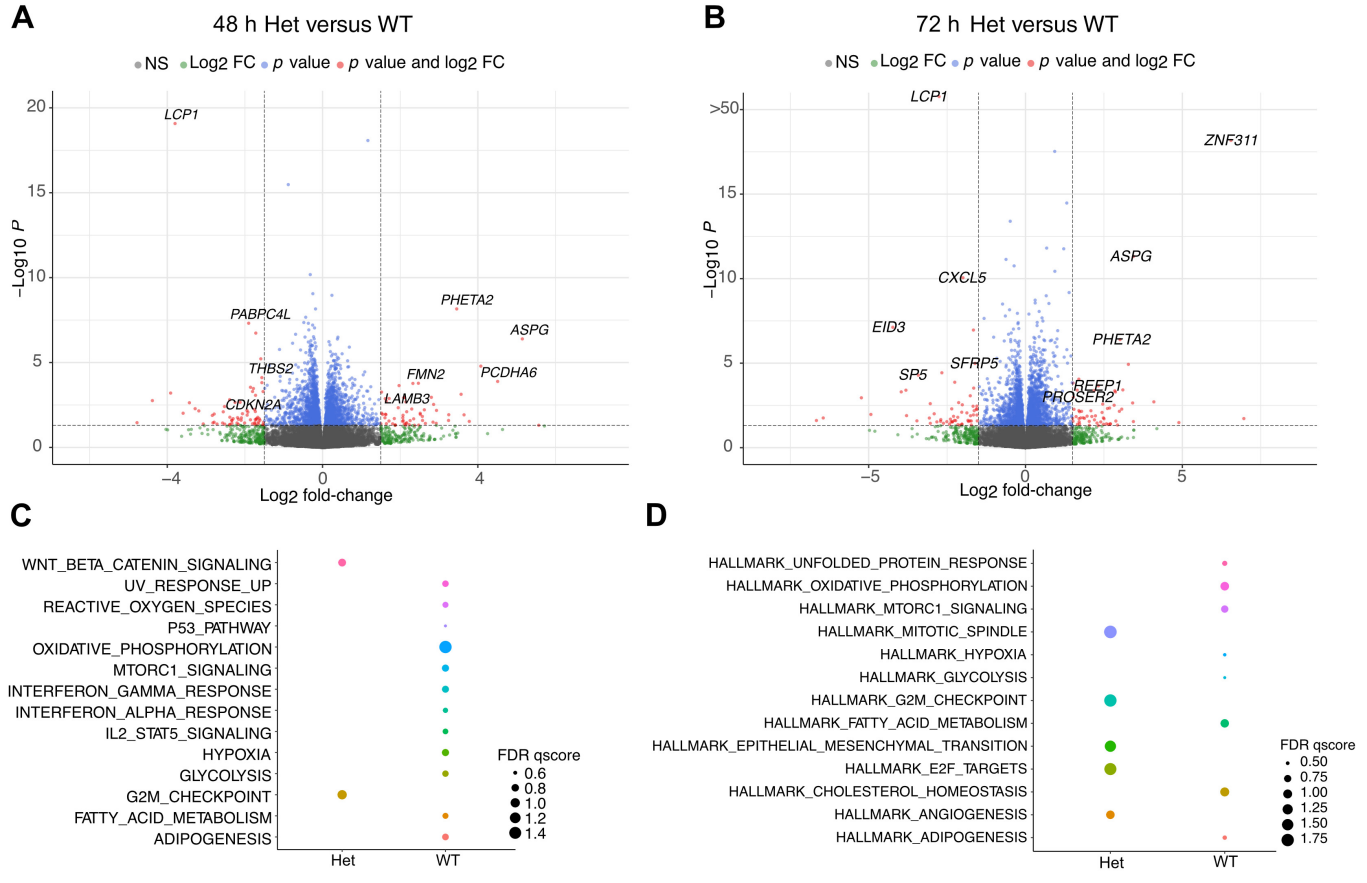
Rs2305089 genotype influences global gene expression. (A,B) Volcano plots showing differentially expressed genes in heterozygous versus WT cell lines at (A) 48 h and (B) 72 h. Log_10_ *p* of <0.05 and Log_2_ fold‐change of >1.5 were considered significant. Genes to the right were upregulated and genes to the left were downregulated in heterozygous cell lines. Each group represents data from three cell lines and three biological replicates, except for WT2, for which two biological replicates were included. (C,D) Dot plot summarising FDR from GSEA of MSigDB hallmark gene sets [39] in heterozygous and WT cell lines at (D) 48 h and (E) 72 h.

We then assessed global gene expression (Figure 3A,B and supplementary material, Data S3). At 48 h, effectors previously implicated in the biology of the notochord and chordoma were among the most differentially expressed in Het cell lines compared to WT. These included *LAMB3* [40] (upregulated), *THBS2* [33] (downregulated), and *CDKN2A* [41] (downregulated). *THBS2*, an extracellular matrix protein expressed in the intervertebral disc, is a notochordal marker [33, 42]. *LAMB3*, a laminin, is required for basement membrane formation during development [40]. *CDKN2A*, a cell cycle regulator, is commonly mutated in chordomas [19, 20]. At 72 h both *SFRP5* and *SP5* were downregulated and are involved in Wnt/β‐catenin signalling [43], which is necessary for the development and maintenance of notochordal cells [44].

The most upregulated and downregulated effectors in the heterozygous lines at 48 h were *PHETA2* and *LCP1*, respectively, both of which are involved in endocytosis and intracellular transport functions, required for the maintenance of notochord vacuoles [45, 46]. A regulator of the endoplasmic reticulum *ERN2/IRE1β* was also identified: the homologue of *ERN1/IRE1α*, which interacts with XBP1, a regulator of notochord formation in Xenopus [47, 48]. *REEP1* regulates lipid droplet formation [49] and was also downregulated. The effectors of migration and apoptosis in other cancers and developing tissues were differentially expressed: *LCP1* [50, 51, 52, 53] and *CXCL5* [54] were upregulated, while *PROSER2* [55] and *PCDHA6* [56] were downregulated.

Next, we utilised GSEA to explore the impact of the variant A allele on the hallmarks of cancer and developmental processes (Figure 3C,D and supplementary material, Figure S3). At 48 h, heterozygous cell lines were enriched for the mitotic spindle and Wnt/β‐catenin pathways (Figure 3C); at 72 h, they were enriched for the G2M checkpoint, mitotic spindle, and E2F target pathways (all associated with proliferation) as well as epithelial mesenchymal transition (EMT), the process by which the notochord is formed [57] and which is associated with *TBXT* expression in epithelial malignancies [58, 59, 60] (Figure 3D). In contrast, WT cell lines were enriched for metabolic pathways, including oxidative phosphorylation, fatty acid metabolism, cholesterol homeostasis, and for MTORC1 signalling (Figure 3C,D).

### The rs2305089 SNP promotes migration without influencing cell morphology, apoptosis, or cell cycle

**Figure 4:**
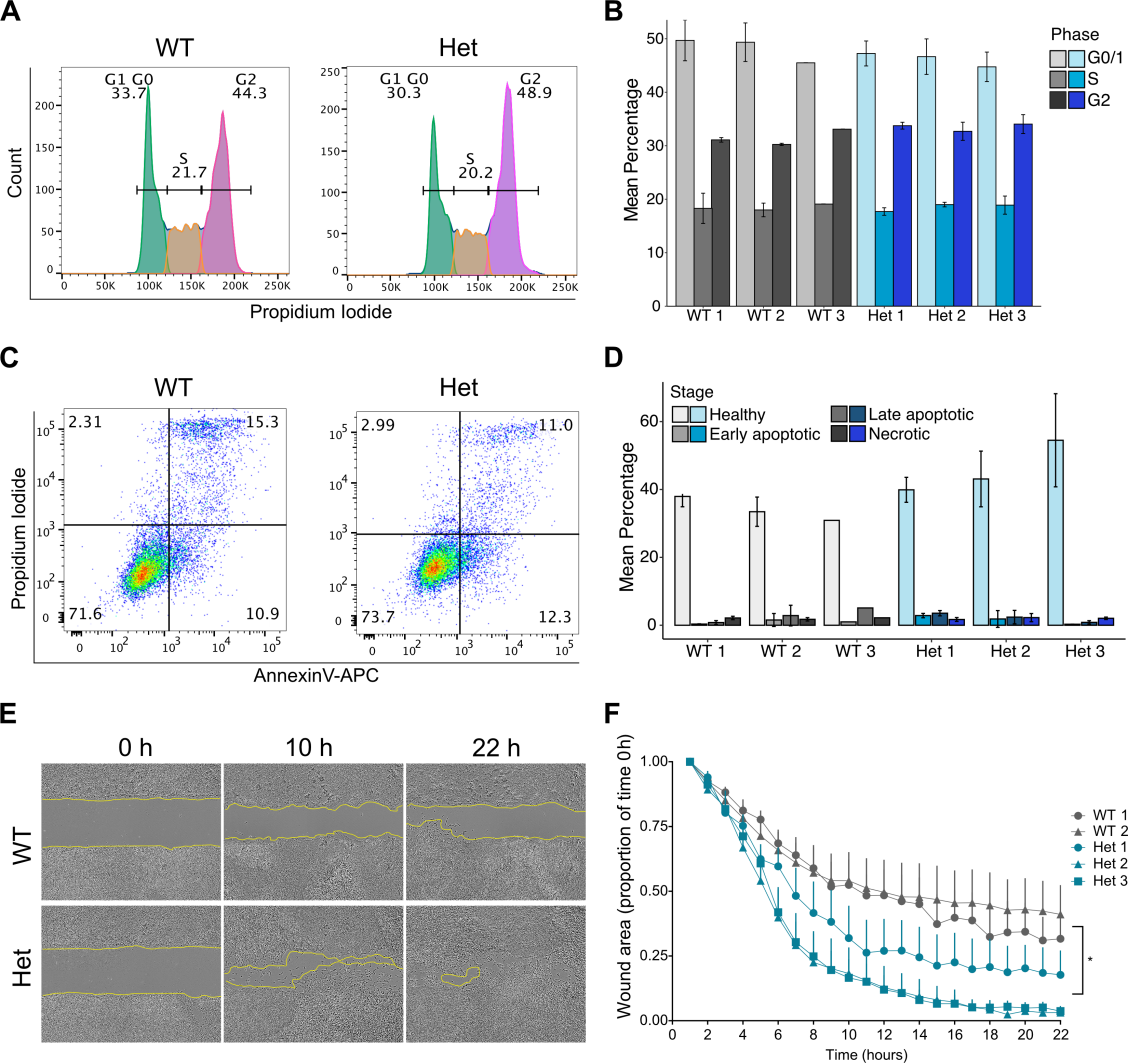
Rs2305089 genotype does not influence cell turnover but modulates migration. The 48‐h time point was chosen for functional assays because this was when TBXT expression was different between the genotypes. (A) Representative FACS histograms of propidium iodide (PI) cell cycle assay during mesoderm phase showing gating strategy and results. WT (*n* = 3) and Het cell lines (*n* = 3) were assessed in duplicate. (B) Histogram summarising proportion of cells in G0/G1, S and G2/M phases for all cell lines in duplicate. *p* = 0.73, two‐way ANOVA. (C) Representative FACS dot plots showing annexin‐V/PI assay. WT and Het cell lines (*n* = 3) were assessed in duplicate. Annexin‐V‐negative/PI‐negative = live cells; annexin‐V‐negative/PI‐positive cells = necrotic cells; annexin‐V‐positive/PI‐positive = late apoptotic cells; annexin‐V‐positive/PI‐negative = early apoptotic cells. (D) Histogram summarising annexin‐V/PI assay results for all cell lines in duplicate. *p* = 0.461, two‐way ANOVA. (E, F) Scratch wound assay during mesoderm phase. Repeated in three independent experiments with five replicates each experiment per cell line. *p* = 0.024, two‐way, repeated‐measurements ANOVA. The plating of WT 3 did not result in a uniform layer in any of the experiments and was therefore excluded. Het = heterozygous. WT = wild type. (E) Representative pictures of bright field scratch assay (4×). (F) Quantification of wound area (as a ratio to the value at time point 0 h) over 22 h. Values are shown as mean ± SEM extending in one direction.

The identification of several oncogenic pathways altered by the rs2305089 A allele led us to investigate its functional impact. Analysis of cell cycle phases and apoptosis levels by flow cytometry (Figure 4A–D) showed no differences between genotypes, suggesting that the rs2305089 SNP did not affect cell turnover. Instead, in line with the enrichment in EMT and the differential expression of genes implicated in migration, we found that heterozygous cell lines showed significantly faster migration compared to the WT cell lines, supporting a role for the rs2305089 SNP in migration during mesoderm commitment (Figure 4E,F).

## Discussion

We demonstrated that BNCTs are more common in patients with chordoma than in the general population and that there is an association between BNCTs and the rs2305089 A variant. Moreover, we find a higher frequency of the AA genotype (48.5%) in BNCTs and also in chordomas (69.2%) compared to controls. Taken together these findings provide further evidence than previously shown that the BNCT is the precursor of chordoma and that the variant A allele predisposes to notochordal tumours. On a clinical note, as multicentric BNCTs were only found in patients with chordoma, we propose that if more than one BNCT is identified on imaging, patients should undergo whole‐spine imaging and prolonged follow‐up.

A limitation of this study is that our data likely under‐represent the prevalence of BNCTs in the general population because they can be extremely small (<1 mm diameter), so some are beyond the limit of detection on MRI scans [8]. Furthermore, BNCTs and chordomas not uncommonly arise in the coccyx, but this anatomical site is not scanned routinely when whole‐spine MRI is undertaken [8]. Conversely, as the diagnosis of BNCTs was based on MRI appearances, the prevalence may be overestimated due to the inclusion of radiological mimics, the closest being the lipid‐poor (radiologically described as ‘atypical’) vertebral haemangioma (VH). Both lesions may show similar signal characteristics and may contain hyperintense T1 foci in keeping with fat. A rim of fat is sometimes seen at the margin of an atypical VH [61] allowing distinction as this appearance is not seen in BNCTs. VHs, although frequently identified in the spine on imaging, are most common in thoracic and lumbar vertebrae, and are rare (<1% of cases) in the sacrum and coccyx [62]: The atypical / lipid‐poor variant will be even rarer at these sites.

Another challenge that we faced was balancing the age and gender in our cohorts but had to consider that chordomas are more common in males. Therefore, we prioritised balancing sex over age. However, including the previously excluded females in the control group did not change the prevalence significantly.

The second component of the study investigated the functional impact of the SNP *in vitro* by developing isogenic iPSC models. The heterozygous genotype was associated with increased expression of *TBXT*, corroborating previous findings [26] and highlighting a potential mechanism in the persistence of notochordal cells. A quarter of people who develop chordomas are heterozygotes for rs2305089 [26, 27], suggesting that a single variant allele contributes to a predisposition to notochordal tumours and likely explains the modest difference in *TBXT* expression between genotypes in our study. One would expect to detect more pronounced differences in *TBXT* expression with the homozygous genotype, the most frequent genotype in patients with chordoma (75%) [26, 27], but it has been demonstrated that the addition of only one A allele has an impact on *TBXT* expression in chordomas [26]. This may be explained by recent research showing that WT TBXT is slightly more stable than the G177D variant protein, raising the possibility that the absence of the WT allele rather than the presence of the A allele accounts for the persistence of the notochord [28].

It is notable that it was not possible to engineer iPSC lines harbouring the homozygous variant. This represents a limitation of our study, but interestingly, the inability to generate homozygous models has been encountered by others [23]. Further work is warranted to address this issue; one option would be to employ iPSCs homozygous for the AA rs2305089 variant and engineering WT alleles into this background.

Our transcriptomic analysis was performed during mesoderm differentiation (48‐h time point), but also at the 72‐h time point. Even though the cells are no longer mesoderm at the latter time point, we demonstrated that there was enrichment of the TBXT network active in chordomas, a finding that can be explained by delayed downstream effects. Our transcriptomic analysis of heterozygous versus WT cell lines revealed the upregulation of *PHETA2* and downregulation of *LCP1*, which are secretory and endoplasmic reticulum effectors, and the differential enrichment of the MTORC1 and metabolic pathways. MTORC1 is recruited and activated at lysosomes [63], which are implicated in the biology of chordomas. Inhibition of GSK‐3Beta, part of the lysosomal signalling pathway, reduces *TBXT* expression and sensitises chordomas to chemotherapy [64]. Failure of notochord vacuole formation and maintenance has been associated with a shortened spine [46] and genes involved in the determination of spine length were identified in our analysis: A human case of *PHETA1* mutation exhibited scoliosis of the spine and other skeletal abnormalities, while *PABPC4L* is associated with height determination in humans [65]. Disruption of XBP1 and TBXT impairs the notochord, causing the lack of a tail in *Ciona* larvae [66]. Recently, an Alu element in *TBXT* was identified as the mechanism for tail loss in human‐primate ancestors [67], and a SNP in *TBXT* was associated with scoliosis in Chinese patients [68]. Together these data support a link between aberrant notochordal biology and phenotypic differences affecting the spine.

Finally, we demonstrated that the heterozygous genotype induced enhanced cellular migration, a hallmark of cancer and a property of stem cells which affects differentiation. This finding echoes the association of *TBXT* expression with migration and metastasis in several carcinomas [58, 59]. Metabolic reprogramming is crucial for differentiation of pluripotent [60, 69] and neural progenitor cells [70] and is supported by the differential enrichment of metabolic pathways in our WT cell lines. Specifically, reprogramming resulting in altered secretory and endoplasmic reticulum effector expression and increased migratory capacity could explain how the rs2305089 SNP impacts the differentiation of notochordal cells during notochord regression predisposing to the development of BNCTs and, ultimately, to chordomas.

## Author contributions statement

AMF and LC conceptualised the study. IU, LC and LL generated the *in vitro* data. POD and IU reviewed MRIs at the Royal National Orthopaedic Hospital. DC reviewed imaging at the National Hospital for Neurology and Neurosurgery. PC advised on the study design and undertook the statistical analysis. DH reviewed MRI scans at University Hospital Basel, Switzerland, and WB reviewed MRI scans at the Royal Prince Alfred Hospital, Sydney, Australia. LC, IU, AMF and POD analysed the data and wrote the manuscript. All authors reviewed the manuscript.

## Supporting information


Supplementary materials and methods
**Figure S1.** Workflow for single nucleotide knock‐in in iPSCs
**Figure S2.** Characterisation of edited clones
**Figure S3.** GSEA for WT and Het clones
**Table S1.** Summary of study groups from radiological study
**Table S2.** Cell lines taken forward for functional study
**Table S3.** List and sequences of primers, guides, and donors used in study
**Data S1.** Representative Sanger sequencing results for 1 kb around rs2305089 variant in *TBXT* of WT clone
**Data S2.** Representative Sanger sequencing results for 1 kb around rs2305089 variant in *TBXT* of Het clone
**Data S3.** Lists of differentially expressed genes (DEGs) from mRNAseq at 48 and 72 h
**Data S4.** Lists of enriched pathways in GSEA

## Data Availability

Raw and processed mRNAseq data have been deposited in the National Center for Biotechnology Information GEO database under GEO accession number GSE277707 (https://www.ncbi.nlm.nih.gov/geo/query/acc.cgi?acc=GSE277707).
