# Supplementary material for: The TBXT rs2305089 SNP links the benign notochordal cell tumour and chordoma
Source: J Pathol. 2025 May 5;266(3):247–57. doi: 10.1002/path.6427 (PMC12146811; doi:10.1002/path.6427)
Supplement: Supplementary file 1 — Supplementary materials and methods Figure S1. Workflow for single nucleotide knock‐in in iPSCs Figure S2. Characterisation of edited clones Figure S3. GSEA for WT and Het clones Table S1. Summary of study groups from radiological study Table S2. Cell lines taken forward for functional study Table S3. List and sequences of primers, guides, and donors used in study Data S1. Representative Sanger sequencing results for 1 kb around rs2305089 variant in TBXT of WT clone Data S2. Representative Sanger sequencing results for 1 kb around rs2305089 variant in TBXT of Het clone Data S3. Lists of differentially expressed genes (DEGs) from mRNAseq at 48 and 72 h Data S4. Lists of enriched pathways in GSEA [file PATH-266-247-s001.docx]

**The *TBXT* germline rs2305089 SNP is associated with benign notochordal cell tumour and chordoma**

I Usher *et al. J Pathol* <https://doi.org/10.1002/path.6427>

**Supplementary materials and methods**

**Supplementary Figures S1–S3**

**Supplementary Tables S1–S3**

**Supplementary Data S1, S2** (provided as a separate PDF file)

**Supplementary Data S3, S4** (provided as separate Excel files)

Reference numbers refer to the main text list

**Supplementary materials and methods**

Sample size calculation

The expected VAF in chordoma is 0.75 [26] to 0.86 [27] (on a total of *n* = 163 patients from the two publications) so was assumed to be 0.80 in the BNCT group. The VAF was expected to be 0.50 in the control group. Using a significance level of 0.05 and a power of 80% for a chi-squared test, 27 would be required to detect a difference of ~0.30 in the VAF between the BNCT and the control groups.

TaqMan genotyping of rs2305089 locus

Buccal swabs and whole blood samples were stored at –80 °C until germline DNA was extracted. The Isohelix Buccal-Prep Plus DNA Isolation Kit (Isohelix, Cell Project Ltd., Kent, United Kingdom; BPP-50) and the QIAamp DNA Blood Mini Kit (Qiagen, 51106) were used for buccal swabs and whole blood extraction respectively, according to the manufacturer’s instructions. TaqMan genotyping was performed using the C11223433_10 assay diluted using nuclease-free water following the manufacturer’s protocol (Thermo Fisher Scientific, 4351379). The reaction mixture included 5 μl TaqMan genotyping mastermix, 0.5 μl C11223433_10 assay, 2.5 μl water, and 2 μl DNA at a concentration of 5–50 ng/μl in 10 μl. The assay was run on a QuantStudio™ qPCR Systems machine, and results were analysed using the Genotyping application on the Thermo Fisher Connect™ cloud.

iPSC-derived mesoderm-like cell cultures

iPSCs were used between passages 40 and 75. iPSCs were expanded in six-well plates coated with Geltrex™ LDEV-Free Reduced Growth Factor Basement Membrane Matrix (A1413202, Gibco Life Technologies), 1:100 in DMEM (31966021, Gibco Life Technologies) using Essential 8™ Flex Medium Kit (E8 Flex, A2858501, Gibco Life Technologies). Cells were passaged twice weekly (1:3 to 1:6) when 80–90% confluent, using 0.5 mM EDTA in Dulbecco’s PBS (14190250, Gibco Life Technologies). Mesoderm differentiation was undertaken following two or three passages after thawing.

CRISPR/Cas9 genetic editing

For steps involving high cellular stress, such as single-cell dissociation and colony picking, E8 Flex medium with RevitaCell™ supplement (Thermo Fisher Scientific) was used for 2 h prior to cell collection and was continued until colony formation. In all other steps, E8 Flex was used without RevitaCell™.

All CRISPR/Cas9 components were purchased from Integrated DNA technologies (IDT, Inc, Coralville, IA, USA). Single-stranded oligodeoxynucleotides (ssODN) were ordered as Alt-R™ HDR Donor Oligos (IDT). The gRNA was prepared by duplexing Alt-R® CRISPR-Cas9 crRNA and Alt-R® CRISPR-Cas9 tracrRNA (IDT, 1072532). The ribonucleoprotein (RNP) was formed using 0.78 μl Alt-R® CRISPR-Cas9 gRNA, 1.02 μl Alt-R® S. p. Cas9 Nuclease V3 (IDT, 1081058), and 1.2 μl PBS (total 3 μl). Cells were detached, counted, and transfected using the Lonza™ P3 Primary Cell 4D-Nucleofector™ X (Lonza, V4XP-3024) and electroporation program CA137. To prepare the electroporation mixture, 0.5×10^6^ cells were resuspended in 20 μl Lonza electroporation buffer, 1 μl RNP complex, and 0.5 μl Alt-R™ HDR Donor Oligo. Following transfection, cells were recovered with Alt-R® CRISPR-Cas9 HDR enhancer (IDT, 1081072) in E8 Flex without antibiotics at 32 °C for 24 h, then enhancer was removed, and cells were cultured at 37 °C in normal conditions.

Once 80–90% confluent the cells were detached, counted and plated in a Geltrex coated 96-well plate (650 cells per well). Once 80–90% confluent, each well was split into a “mirror” plate containing cells from which DNA was extracted and a plate that was maintained in culture. To this end, the well was washed with 100 μl DPBS and detached with 30 μl Accutase (Thermo Fisher Scientific), collected with 70 μl of E8 Flex and 50 μl were transferred to a mirror 96-well plate and stored at −80 °C, whereas the remaining 50 μl were seeded for colony picking.

DNA extraction from mirror plate

The plate was spun at 1,950 RCF for 30 min at 4 °C, the medium was removed, and the plate stored at −80°C. Upon thawing, 30–50 μl of Lucigen QuickExtract™ DNA Extraction Solution (LGC, Middlesex, United Kingdom, QE09050) was added, and the mixture was triturated and heated at 65 °C for 6 min then 98 °C for 2 min and used directly for genotyping using droplet digital PCR (ddPCR) (see below).

Colony picking

Populations for which we identified the highest editing rates by ddPCR were taken forward for colony picking. Between 750 and 1,000 cells were seeded sparsely into a 10-cm plate so that colonies expanded from single cells and grew apart from each other. The individual colonies were expanded until they were of sufficient size for colony picking. Colonies were visualised under an inverted microscope at a magnification of ×10 to ×20, and the colony was scraped and aspirated under direct vision using a 200-μl pipette set to a volume of 100 μl. The picked colony was placed into a 96-well plate containing 50 μL E8 Flex with 1X Revitacell and triturated 8–10 times to break up the colony. When cells were 50–70% confluent, they were split 1:2, half were seeded for genomic DNA extraction and half for subculture or freezing.

Sanger sequencing and ddPCR

For genotyping, DNA of picked colonies in 96-well mirror plates was extracted using Lucigen QuickExtract™ DNA Extraction Solution (LGC, QE09050). DNA was extracted using Zymo Column Extraction (Zymo Research, Irvine, CA, USA, D3024) from established expanded cell lines.

ddPCR

A common primer set and probes for each allele were designed: a hexachlorofluorescein (HEX) probe for the parental allele (A/T) and fluorescein amidite (FAM) probe for the edited allele (G/C). Primers and probes are listed in supplementary material (Table S2). The BioRad QX200 ddPCR supermix for probes (no dUTP) workflow, Automated Droplet Generator, BioRad Automated Droplet Generation Oil for Probes (BioRad, Hercules, California, USA; Catalogue No.: 1864110), Eppendorf vapo.protect thermocycler, and QX200 Automated Droplet Reader were used. Results were analysed using the BioRad QuantaSoft™ Analysis Pro Software using the rare event detection setting.

Sanger sequencing

PCR was performed using primers listed in supplementary material (Table S2). The reaction contained 12.5 μl AmpliTaq Gold™ 360 Master Mix (Thermo Fisher Scientific, 4398881), 0.5 μl 10 μM forward-reverse primer mix, 10 μl water plus 2 μl Lucigen QuickExtract™ DNA. PCR products were cleaned using the ExoSAP-IT™ Express PCR Product Cleanup Reagent (Applied Biosystems, Thermo Fisher Scientific, 15563677) and sent for sequencing (Source BioScience, Nottingham, United Kingdom).

Quantitative real-time PCR (qPCR)

iPSCs were subcultured in a 1:25 split into 24-well plates coated with Geltrex and differentiated as described previously [32]. At the indicated time point, cells were washed in PBS and collected, and RNA was extracted from cell line pellets using the Quick-RNA Miniprep Kit (R1054, Zymo Research Corp.) and reverse transcribed into cDNA using the High Capacity cDNA Reverse Transcription Kit (Applied Biosystems, Thermo Fisher Scientific, Loughborough, Leicestershire, UK) following the manufacturers’ protocols. qPCR was performed using Fast SYBR® Green MasterMix (Applied Biosystems) following the manufacturer’s instructions. Primers are listed in supplementary material (Table S2). For analysis, the means of cycle thresholds (Ct) (*n* = 2 technical replicates per sample per gene) were calculated. The fold increase of *TBXT* over *GAPDH* was determined [=POWER(2,-deltaCt)] based on the deltaCt between *TBXT* and *GAPDH*.

Western blotting for TBXT and protein quantification

iPSCs were subcultured in a 1:25 split into 24-well plates coated with Geltrex and differentiated as described previously [32]. At the indicated time point, cells were washed in PBS and scraped in 200 ul RIPA buffer. Proteins were separated using a 4–15% Crit TGX Stain-Free Gel (Bio-Rad, 5678083) and transferred on a nitrocellulose membrane, which was blocked with 5% BSA (Sigma Aldrich) in T-BST followed by overnight incubation at 4° C with the Brachyury (A-4) antibody (Santa Cruz, sc-374321) 1:1,000 in 5% BSA. The following day the membrane was further incubated with an antibody against beta-actin (Sigma Aldrich, A5441) as a loading control. Blots were scanned using the Odyssey® CLx Imaging System (LI-COR Biotech, LLC, USA). Quantification was performed using Image Studio™ software: Each band was normalised for the background value around the band and quantified relative to the corresponding beta actin after background normalisation.

U-CH1

The U-CH1 chordoma cell line, used as a positive control for *TBXT* expression, was generously provided by the Chordoma Foundation and cultured according to their instructions (https://www.chordomafoundation.org/researchers/disease-models/u-ch1/).

Messenger RNA (mRNA) sequencing

iPSCs were subcultured in a 1:25 split into 24-well plates coated with Geltrex and grown for 12 h, ensuring sufficient space for colonies to grow from single cells. Colonies were recovered overnight in E8 Flex medium, then medium was changed to 500 μl Cardiomyocyte Differentiation Medium A (Thermo Fisher Scientific, A29209-01). At each time point, each well was washed once with 500 µl PBS, and 300 µl TRIzol® reagent (Invitrogen) was added until cells were detached. Then 300 µl 100% ethanol was added to the cell lysate solution. RNA was extracted using the Zymo Direct-zol RNA kit (Zymo R2061), including an on-column DNA digestion following the manufacturer’s protocol. RNA quality control and quantification were undertaken on the Agilent RNA Tapestation 2200 with RNA Screentape (5067-5576): RNA Integrity Number (RIN) measurements were 8.7 to 9.8.

Functional assays

Apoptosis assay

Apoptosis was determined by detecting phosphatidylserine by allophycocyanin (APC)-conjugated annexin V using the APC Annexin V Apoptosis Detection Kit with PI (Biolegend). Cells (200,000) were plated for each cell line and each condition (stained/unstained). Cells were harvested and washed once in PBS and resuspended in 250 μl of binding buffer containing 5 μl annexin V–APC and 10 μl PI solution. Cells were incubated in the dark for 15 min before being analysed on an LSR Fortessa (Becton Dickinson) running FACSDiva Software version 6 with 10^4^ events recorded for each sample.

Cell cycle studies using PI staining

For each cell line, 200,000 cells were plated. Cells were harvested, washed once in PBS (Thermo Fisher Scientific), and counted. Cells were fixed in 70% ethanol in PBS on ice for 30 min. Fixed cells were centrifuged at 3,000 rpm for 5 min and washed with PBS. To ensure that only DNA was stained, the pellet was treated with ribonuclease A (100 μg/ml in PBS, Thermo Fisher Scientific) and subsequently stained with PI solution in PBS (50 μg/ml, Sigma-Aldrich) at room temperature in the dark for 30 min prior to being analysed on an LSR Fortessa (Becton Dickinson) running FACSDiva Software version 6 with 10^4^ events recorded for each sample.

**Supplementary Figures S1–S3**


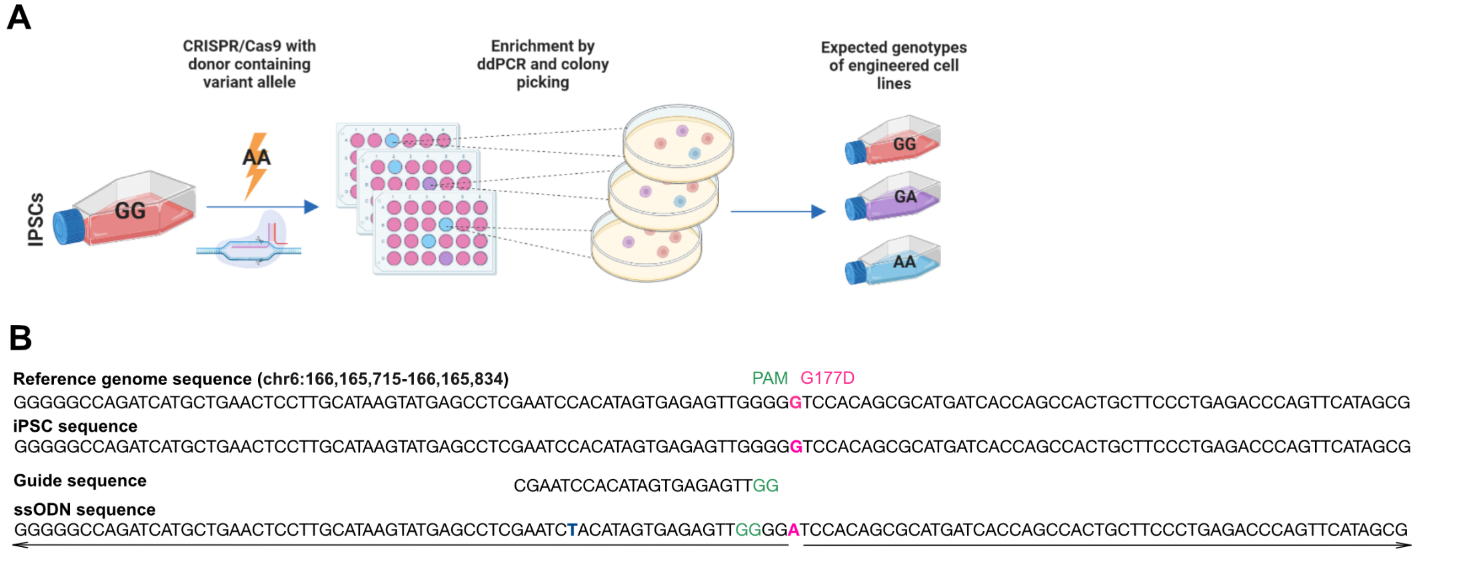


**Figure S1.** Workflow for single-nucleotide knock-in in iPSC. (A) Workflow for early characterisation of CRISPR/Cas9 knock-in edited iPSCs. After transfection with CRISPR/Cas9, cells were seeded in 96-well plates. Wells were screened by ddPCR, and the wells showing an enrichment of edited clones were expanded for colony picking to isolate the clonal line. ddPCR and Sanger sequencing were used to confirm the genotypes of the expanded cell lines, created with BioRender.com. (B) Design of CRISPR/Cas9 components targeting rs2305089 SNP (G177D amino acid change) in TBXT in iPSCs. Blue “T” in ssODN donor sequence indicates modification to prevent Cas9 cleavage of donor. PAM = protospacer adjacent motif.


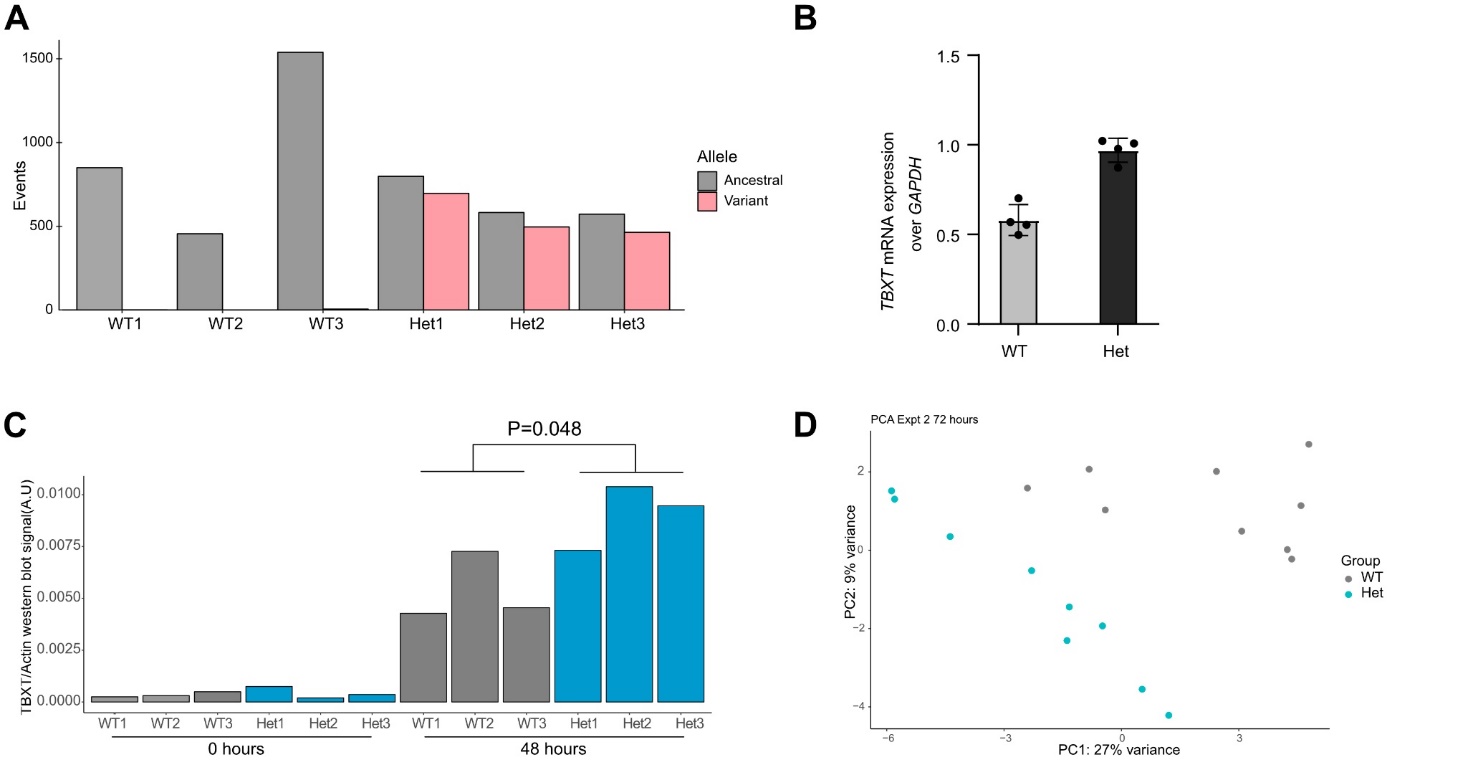


**Figure S2.** Characterisation of edited clones. (A) Results of ddPCR assay for rs2305089 allele expression performed on cDNA of WT and Het cells differentiated for 48 h. Events = no. droplets detected by each probe. Het cell lines show expression of both alleles (G = ancestral, A = variant) in roughly equal proportions, whereas WT cell lines showed expression of only the ancestral G allele, *n* = 3 per cell line. (B) *TBXT* gene expression determined using RT-qPCR in WT and Het clones in an independent experiment further to those that underwent mRNAseq with four biological replicates. (C) Quantification of TBXT western blot shown in Figure 2D. The ratio of band intensity of TBXT over beta actin is shown for WT and Het samples; *p* = 0.048, Welch two-sample *t*-test between replicates WT and Het samples. (D) PCA plot of all mRNAseq data at 72 h. Cell lines (*n* = 3 per genotype) and replicates (*n* = 3 per cell line) have been collapsed into genotypes.

**
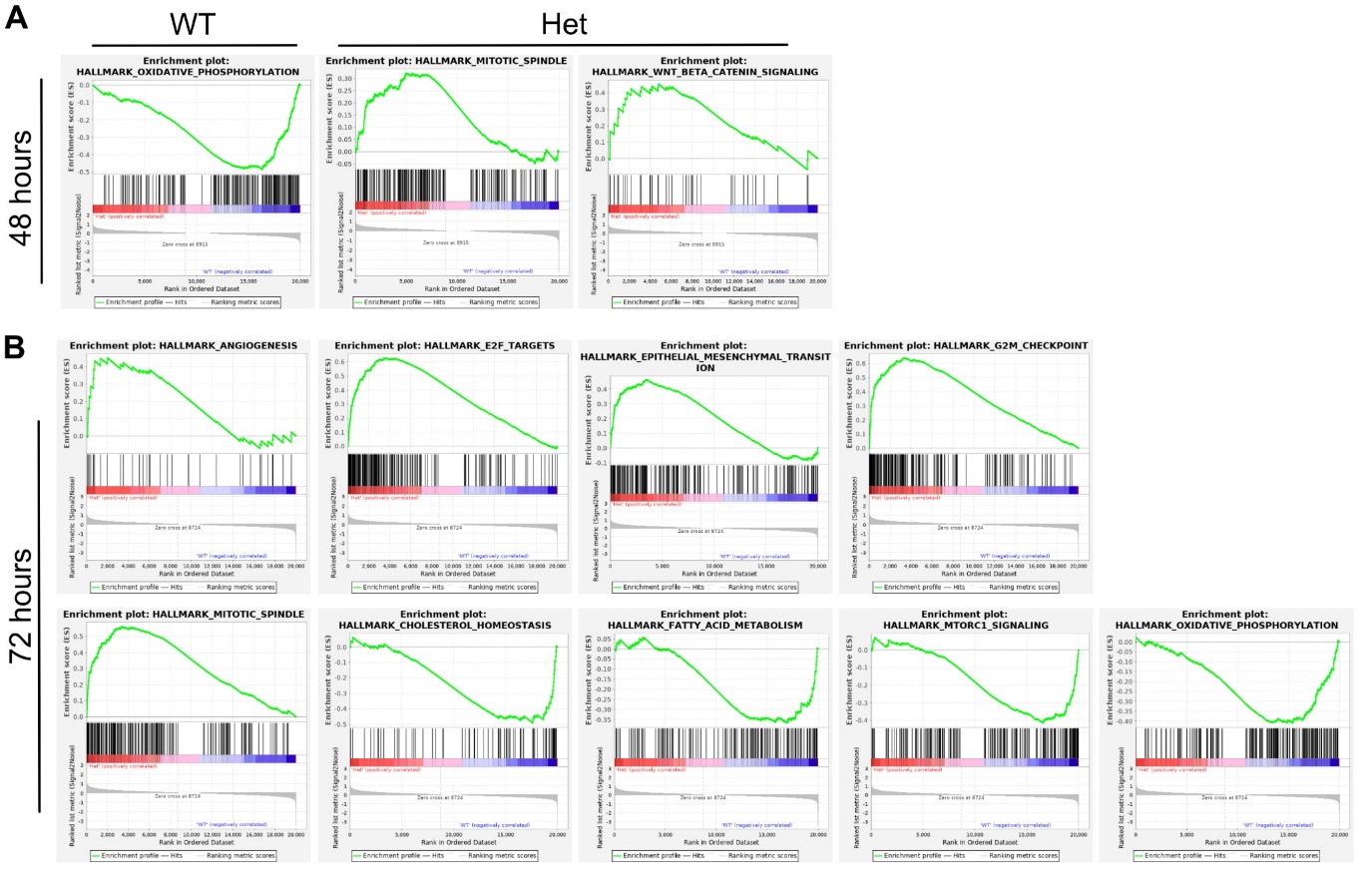
**

**Figure S3.** GSEA for WT and Het clones. Enrichment plots of top hits pathways identified in GSEA transcriptomic analysis at (A) 48 h and (B) 72 h.

**Supplementary Tables S1–S3**

**Table S1.** Summary of study groups from radiological study.

|  | **Control** | **Chordoma** | ***P* value** |
| --- | --- | --- | --- |
| No. patients | 74 | 109 | - |
| Age in years at presentation range (mean) | 20–84 (47) | 14–90 (59) | <0.001 |
| White European ethnicity (%) | 52 (70) | 92 (84) | 0.674 |
| Female patients (%) | 23 (31) | 33 (30) | 0.908 |

**Table S2.** Cell lines taken forward for functional study.

| **Clone** | **Name** | **Type** | **Genotype** |
| --- | --- | --- | --- |
| 4610N1 | WT 1 | Wild type (unedited) | CC/GG |
| 4610N2 | WT 2 | Wild type (unedited) | CC/GG |
| 4610N3 | WT 3 | Wild type (unedited) | CC/GG |
| 463N1 | Het 1 | Heterozygous | CT/GA |
| 463N2 | Het 2 | Heterozygous | CT/GA |
| 463N3 | Het 3 | Heterozygous | CT/GA |

**Table 3.** List and sequences of primers, guides, and donors used in study.

| **Application** | **Name** | **Nucleotide sequence** |
| --- | --- | --- |
| qPCR | *TBXT Fw* | CCCGTCTCCTTCAGCAAAGTC |
| qPCR | *TBXT Rev* | TGGTGAAGACGCCAGTGGA |
| Sanger sequencing | Fw primer | TTCAGTGCCACCAATCCTGTAT |
| Sanger sequencing | Rev primer | CACTTGTATGGAGAATTCAAGG |
| Sanger sequencing | 1 Kb around TBXT Fw | CGTCTGCCCTGGAGAAACT |
| Sanger sequencing | 1 Kb around TBXT Rev | AGTTCTCTCCTGTGCTTCCA |
| ddPCR | Forward primer | GCCACCAATCCTGTATC |
| ddPCR | Reverse primer | TCAGGGAAGCAGTGG |
| ddPCR | Variant allele probe | [HEX]TCATGCGCTGTGGATCC[BHQ1] |
| ddPCR | Wild type allele probe | [6FAM]TCATGCGCTGTGGACCC[BHQ1] |
| CRISPR component | Guide RNA 1 | CGAATCCACATAGTGAGAGTTGG |
| CRISPR component | Single-stranded oligonucleotide  donor template | TCAACGGAGGGGGCCAGATCATGCTGAACTCCTTGCATAAGTATGAGCCTCGAATCTACATAGTG AGAGCTTTGGGGATCCACAGCGCATGATCACCAGCCACTGCTTCCCTGAGACCCAGTTCATAG |

FAM, fluorescein amidite; HEX, hexachlorofluorescein; BHQ, black hole quencher; ddPCR, droplet digital PCR.
